# Australian English listeners' perception of Japanese vowel length reveals underlying phonological knowledge

**DOI:** 10.3389/fpsyg.2023.1122471

**Published:** 2023-10-26

**Authors:** Kakeru Yazawa, James Whang, Paola Escudero

**Affiliations:** ^1^Institutes of Humanities and Social Sciences, University of Tsukuba, Tsukuba, Japan; ^2^Department of Linguistics, Seoul National University, Seoul, Republic of Korea; ^3^The MARCS Institute for Brain, Behaviour and Development, Western Sydney University, Sydney, NSW, Australia

**Keywords:** Australian English, Japanese, cross-linguistic perception, vowel, phonological feature, length, acoustic cue, duration

## Abstract

Speech perception patterns are strongly influenced by one's native phonology. It is generally accepted that native English listeners rely primarily on spectral cues when perceiving vowels, making limited use of duration cues because English lacks phonemic vowel length. However, the literature on vowel perception by English listeners shows a marked bias toward American English, with the phonological diversity among different varieties of English largely overlooked. The current study investigates the perception of Japanese vowel length contrasts by native listeners of Australian English, which is reported to use length to distinguish vowels unlike most other varieties of English. Twenty monolingual Australian English listeners participated in a forced-choice experiment, where they categorized Japanese long and short vowels as most similar to their native vowel categories. The results showed a general tendency for Japanese long and short vowels (e.g., /ii, i/) to be categorized as Australian English long and short vowels (e.g., /i:, ɪ/ as in “heed,” “hid”), respectively, which contrasts with American English listeners' categorization of all Japanese vowels as tense regardless of length (e.g., /ii, i/ as both “heed”) as reported previously. Moreover, this duration-based categorization was found not only for Australian English categories that contrast in duration alone (e.g., /ɐ:, ɐ/ as in “hard,” “hud”) but also for those that contrast in both duration and spectra (e.g., /o:, ɔ/ as in “hoard,” “hod”), despite their spectral mismatch from the corresponding Japanese vowels (e.g., /aa, a/ and /oo, o/). The results, therefore, suggest that duration cues play a prominent role across all vowel categories—even nonnative—for Australian English listeners. The finding supports a feature-based framework of speech perception, where phonological features like length are shared across multiple categories, rather than the segment-based framework that is currently dominant, which regards acoustic cues like duration as being tied to a specific native segmental category. Implications for second and foreign language learning are discussed.

## 1. Introduction

Languages differ as to which acoustic cues are phonologically meaningful and in what way. Some languages such as Arabic, Czech, Japanese, and Swedish utilize vowel duration phonemically (International Phonetic Association, 1999), where long and short vowels of the same quality convey different lexical meanings (e.g., *ii* [i:] “good”—*i* [i] “stomach” in Japanese).^1^ A few languages such as Estonian even employ a more complex, three-way distinction (Asu and Teras, 2009): *kalu* [kɑlu] “fish” (partitive plural)—*kaalu* [kɑ:lu] “scales” (genitive singular)—*kaalu* [kɑ::lu] “scales” (partitive singular). English, on the other hand, is said to lack phonological vowel length, since changes in vowel duration alone would not change the meaning of the word (e.g., *Do it!* [du ɪt]—*Doooo iiiit!* [du: ɪ:t]). Such cross-linguistic differences in native phonology are known to shape speech perception patterns (Jacquemot et al., 2003; Escudero et al., 2009; Mazuka et al., 2011; Lipski et al., 2012; Yazawa et al., 2020). For instance, it is generally accepted that English listeners rely primarily on spectral cues and little on duration cues when perceiving native and nonnative vowels because vowel length is not phonemic in English (Hillenbrand et al., 2000; McAllister et al., 2002; Hirata, 2004; Dietrich et al., 2007; Kondaurova and Francis, 2008; Nishi et al., 2008; Mugitani et al., 2009; Karpinska et al., 2015). The literature on vowel perception by English listeners, however, shows a marked bias toward American English (AmE), with the phonological diversity among different varieties of English being largely overlooked. The current study therefore examines the perception of Japanese vowel length by native listeners of Australian English (AusE), which is reported to use length to distinguish vowels unlike most other varieties of English.

Previous research on AmE listeners has found a marginal role of duration as a perceptual cue for vowel identity. Hillenbrand et al. (2000) tested native AmE listeners on synthesized /hVd/ tokens with altered vowel durations, finding a small overall effect of duration on their vowel identification. While some vowel contrasts such as /ɛ/-/æ/ and /ʌ/-/ɑ(ɔ)/ were significantly affected by duration, those that differ systematically in duration such as /i/-/ɪ/, /u/-/ʊ/, /ɪ/-/e/-/ɛ/ were minimally affected. Similarly, Kondaurova and Francis (2008) used synthetic *beat*-*bit* tokens varying in nine perceptually equidistant spectral and durational steps, finding that AmE listeners relied predominantly on vowel spectra. The primacy of spectral cues has also been found in cross-linguistic and second language (L2) perception by AmE listeners. Of particular relevance to the current study, Nishi et al. (2008) found that AmE listeners categorized Japanese long and short vowels (embedded in /hVba/, spoken by four male Japanese speakers in citation and sentence forms) as most similar to AmE tense vowels regardless of length (Table 1). The duration of the Japanese vowels was thus being ignored, although a small effect of duration was found in the categorization of Japanese /ee/-/e/ and /aa/-/a/, possibly reflecting the status of AmE /ɛ/-/æ/ and /ʌ/-/ɑ(ɔ)/ discussed above. Hirata (2004) further tested whether first-language (L1) AmE listeners can learn to correctly identify Japanese vowel length contrasts through supervised perceptual training. The result showed a statistically significant improvement from pre-test (overall 39% correct) to post-test (about 54% correct for the sentence condition and 80% correct for the isolated condition), indicating that the length contrasts are difficult yet learnable for AmE listeners. Finally, the observed underutilization of vowel duration by AmE listeners is an influence of native phonology, as AmE-learning 18-month-old infants can detect changes in vowel duration in the same way as Japanese adults but do not interpret the changes as lexically contrastive (Dietrich et al., 2007; Mugitani et al., 2009).

**Table 1:** Categorization of Japanese vowels by AmE listeners (in percentage, bold = modal responses).

		**Japanese vowel stimuli**									
		**/ii/**	**/i/**	**/ee/**	**/e/**	**/aa/**	**/a/**	**/oo/**	**/o/**	**/uu/**	**/u/**
Perceived AmE vowel	/i/	**99**	**95**								
		/ɪ/	1	4	2	16						
		/eɪ/			**94**	**76**						
		/ɛ/		1	5	8						
		/æ/					2	3				
		/ɑ(ɔ)/					**89**	**57**				
		/ʌ/					9	39		1	1	3
		/oʊ/							**99**	**95**	2	1
		/u/							1	2	**92**	**91**
		/ʊ/								1	5	5

Adapted from Nishi et al. (2008), citation condition.

Much less is understood about listeners of other varieties of English, which warrants attention since different varieties of a language can exhibit divergent perceptual patterns (Escudero and Boersma, 2004; Escudero and Williams, 2012; Escudero et al., 2012; Williams and Escudero, 2014). Again using synthetic *beat*-*bit* tokens differing in spectral and duration steps, Karpinska et al. (2015) found that English listeners from England, Scotland, Wales, Ireland, New Zealand, and Singapore behaved similarly to AmE listeners, showing primary reliance on vowel spectra. Thus, it appears that listeners of most varieties of English are perceptually alike, i.e., underutilizing duration for vowel identity. However, the study also found a distinct perceptual pattern in AusE listeners, who relied primarily on duration rather than spectra. Williams et al. (2018) extended this finding by showing that duration, along with vowel inherent spectral change (VISC), is a crucial cue for AusE listeners to distinguish *bid* from *bead* and *beard*. This makes AusE listeners an exception, at least regarding high front vowels. Chen et al. (2014) also found that AusE-learning 18-month-olds perceive the durational difference between AusE /ɐ:/ and /ɐ/ as lexically contrastive, suggesting that the duration-based perception extends to non-high-front vowels. AusE listeners' sensitivity to vowel duration has been documented in their nonnative perception as well. Tsukada (2012) conducted an AXB discrimination test of vowel length contrasts in Arabic (/ii, aa, uu/—/i, a, u/) and Japanese (/ii, ee aa, oo, uu/—/i, e, a, o, u/) by native Arabic, Japanese, and AusE listeners, where the Arabic and Japanese groups were expected to outperform the AusE “control” group in nonnnative perception because “the extent to which vowel duration is used contrastively in Australian English is likely to be more limited than in Arabic or Japanese” (Tsukada, 2012 p. 511). Contrary to the expectation, the study found no significant advantage of Arabic and Japanese listeners over AusE listeners, who achieved an overall discrimination accuracy of 82% for Arabic vowels and 75% for Japanese vowels despite both languages being nonnative. This, in turn, indicates that AusE listeners are generally sensitive to vowel duration.

The results of Tsukada (2012) show that AusE listeners are able to use duration to discriminate nonnative Japanese vowels. The current study further tests how AusE listeners identify Japanese long and short vowels as their native categories in a forced-choice perception experiment. The distinction between discrimination and identification is important here, since the ability to detect changes in acoustic-phonetic vowel duration does not entail that length is part of phonological vowel identity, as the aforementioned studies on AmE-learning infants have demonstrated (Dietrich et al., 2007; Mugitani et al., 2009). Unlike AmE listeners whose categorization of Japanese vowels was largely unaffected by length (Table 1), AusE listeners may categorize Japanese long and short vowels into different AusE categories according to length (Table 2).^2^ If so, this would indicate that length determines phonological vowel identity in AusE, making it an exception among the many varieties of English thought to lack contrastive length.

**Table 2:** AusE vowel categories and example words (Cox and Palethorpe, 2007).

**Vowel**	**Word**	**Vowel**	**Word**
/i:/	*heed*	/ɪ/	*hid*
/e:/	*haired*	/e/	*head*
/ɜ:/	*heard*	/æ/	*had*
/ɐ:/	*hard*	/ɐ/	*hud*
/o:/	*hoard*	/ɔ/	*hod*
/ʉ:/	*food*	/ʊ/	*hood*
/ɪə/	*feared*

A theoretically important question pertinent to the above prediction is whether AusE listeners' use of duration in vowel categorization would vary depending on the type of Japanese vowel. In AusE, only a subset of vowel categories contrast in duration alone (/e:/-/e/ and /ɐ:/-/ɐ/), while others contrast in both duration and spectra (Cox, 2006; Cox and Palethorpe, 2007; Ratko et al., 2022). It is thus possible that AusE listeners more readily use duration when perceiving Japanese vowels that spectrally match the former (e.g., /ee/-/e/ and /aa/-/a/) than those matching the latter (e.g., /ii/-/i/). Alternatively, given their general sensitivity to vowel duration in nonnative length discrimination (Tsukada, 2012), AusE listeners may equally utilize duration in categorizing all Japanese vowels. These two possibilities are closely related to segment- and feature-based frameworks of speech perception. Current models of cross-linguistic perception generally subscribe to the segment-based view. For example, the Perceptual Assimilation Model (PAM; Best, 1995; Best and Tyler, 2007) and the Speech Learning Model (SLM; Flege, 1995; Flege and Bohn, 2021) explain cross-linguistic perception patterns as a result of nonnative sounds being assimilated to or classified as equivalent to existing native segmental categories. A common implicit premise of these models is that the use of acoustic cues in the assimilation or classification process is specific to each native category. Thus, if duration is an important cue for certain categories but not for others in the L1, then the categorization of nonnative sounds assimilated to the former categories will be duration-dependent and that of those assimilated to the latter categories will not be. The alternative, feature-based view derives from the “feature” hypothesis, which asserts that “L2 features not used to signal phonological contrast in L1 will be difficult to perceive for the L2 learner” (McAllister et al., 2002, p. 230). A crucial assumption underlying this view is that a feature is available to the whole phonological system, independent of specific categories. Thus, if a length feature exists in L1 phonology, then the use of duration cues owing to the feature may extend beyond certain L1 categories with the feature.

Previous studies of cross-linguistic length perception with other languages provide mixed evidence for the above two frameworks. Chládková et al. (2013) examined pre-attentive sensitivity to duration in native and nonnative vowels across Dutch, Czech, and Spanish, using electroencephalography (EEG) to measure mismatch negativity (MMN). Dutch was of particular interest because its phonological status of vowel duration is rather unclear, with all vowel categories that contrast in duration also contrasting in spectra (e.g., /a:/ in *maan* “moon”—/ɑ/ in *man* “man”). It was found that Dutch listeners' sensitivity to duration was comparable to Czech listeners' but greater than Spanish listeners' when the vowel quality was [a] (i.e., native to all three languages), suggesting that Dutch uses vowel duration phonemically as in Czech. However, Dutch listeners' sensitivity to duration was significantly reduced compared to Czech listeners when the quality was changed to Estonian [ɤ] (i.e., nonnative to all three languages). This suggests that Dutch listeners do not disentangle duration cues from spectral cues, perhaps due to their obligatory co-occurrence, consequently confining the phonemic use of vowel duration to native vowel categories. While the result needs to be treated with caution because no significant difference in MMN was found within Dutch listeners between the native and nonnative conditions, the overall finding aligns with the segment-based view. Chládková et al. (2015b) further examined Dutch listeners' perceptual sensitivity to duration in [a] and [ɑ] qualities and found a larger MMN amplitude for the former. This suggests that duration is phonemically relevant for the *maan* vowel that is represented as “long” but phonemically unspecified for the *man* vowel, providing further evidence that the use of duration is vowel-specific in Dutch.^3^

In contrast, the aforementioned study of McAllister et al. (2002) lends support to the feature-based view. The study compared the perception and production accuracy of L2 Swedish vowel length by L1 Estonian, AmE, and Spanish participants, who had all lived in Sweden for at least 10 years. It was found that the L1 AmE and Spanish groups performed significantly worse than the L1 Estonian group, which was taken as evidence for the transfer of a vowel length feature that is present in Estonian but is absent in AmE or Spanish. Of particular note from the results is that the Estonian group was indistinguishable from native Swedish controls in their implementation of duration during production. In Swedish, the length of vowels and consonants are in complementary distribution in stressed syllables—a short consonant follows a long vowel and a long consonant follows a short vowel—which differs from Estonian where vowels and consonants have independent length. The results, therefore, suggest that Estonian speakers were able to learn and implement the complementary duration for consonants and vowels in L2 Swedish, despite no such relationship existing in their L1. Adding to this finding, Pajak and Levy (2014) found that native listeners of a language with vowel length contrasts showed enhanced discrimination of nonnative consonant length contrasts (i.e., geminates). These results together imply the existence of a length feature that is shared across vowel and consonant categories, which seems accessible in nonnative and L2 perception. However, given that the above two studies focus on perception accuracy while those in support of the segment-based view (Chládková et al., 2013, 2015b) focus on perceptual sensitivity, these sets of evidence may not be strictly compatible with each other, thus leaving room for further investigation.

Following the discussion above, the predictions going into the current study are summarized as follows. First, if vowel length is indeed used phonologically in AusE, AusE listeners will show a tendency to categorize long and short Japanese vowels as long and short AusE counterparts, respectively. Second, should the results show stronger duration effects for certain Japanese vowels (e.g., /ee/-/e/ and /aa/-/a/), this would suggest that AusE listeners utilize duration cues only as necessitated by their native categories (e.g., /e:/-/e/ and /ɐ:/-/ɐ/), supporting the segment-based framework of speech perception. Alternatively, should similar duration effects be observed across all vowel qualities, this would suggest that AusE listeners are able to extend the use of duration cues beyond their native categories, supporting the feature-based framework. These predictions will be tested in the experiment presented below.

## 2. Materials and methods

### 2.1. Participants

Twenty female native AusE listeners were recruited for the experiment at Western Sydney University, Sydney, Australia. They were undergraduate or graduate students at the University between the ages of 17 and 35 (mean age = 21.4), born and raised in the greater Sydney area. All participants reported normal hearing and only very basic knowledge of any foreign language. They were compensated for their time in the form of course credit.

### 2.2. Stimuli

The stimuli were 10 Japanese vowels—five long /ii, ee, aa, oo, uu/ and five short /i, e, a, o, u/—embedded in three consonantal contexts (/bVp, dVt, gVk/) and spoken by 10 native Japanese speakers (five female, five male), for a total of 300 tokens. These are a subset of the production data reported in Yazawa and Kondo (2019). The speakers were students or graduates of universities in Japan between the ages of 21 and 27 (mean age = 23.9) who had spent most of their lives in Tokyo and surrounding areas. They read aloud the sentence */CVCe/—/CVCo/—/*CVCe*/ to /CVCo/ ni wa V ga aru* “/CVCe/—/CVCo/—In /CVCe/ and /CVCo/ there is V,” where V is the target Japanese vowel with the lexical pitch accent.^4^ Each sentence was presented in Japanese *kana* orthography, which the speakers read at a comfortable speed. The /e/ in the underlined /CVCe/ was then excised at the first positive zero crossing of the vowel to create /CVC/ stimuli in Praat (Boersma and Weenink, 2022). The utterances were recorded in an anechoic chamber at Waseda University, Tokyo, Japan, using a SONY F-780 microphone with a 44,100 Hz sampling frequency and 16-bit resolution. The volume of all stimuli was adjusted to have a peak intensity of 70 dB.

### 2.3. Procedure

Prior to the experiment, the participants signed a consent form and completed a language background questionnaire. They were informed that they would be listening to “sounds from a foreign language.”

The experiment was a forced-choice task, where the participants had to categorize the vowel in the aforementioned Japanese /CVC/ stimuli presented in isolation. During the experiment, the participants were shown on a computer monitor a list containing the words in Table 2. After hearing each stimulus, the participants chose the word containing a vowel that best matched the vowel in the stimulus (e.g., [di:t] → *heed*). The words in the list all had the shape /hVd/, except for two words that had the shape /fVd/. Participants were asked to make their choice as quickly as possible. The stimuli were presented in random order through noise-isolating headphones, and participants responded by clicking the word choice with a computer mouse. A break was programmed to occur after 150 tokens (i.e., midpoint of experiment), which ended when participants clicked the mouse. The experiment was conducted in a sound-attenuated room at Western Sydney University, using PsychoPy2 (Peirce, 2007), which recorded participants' responses and response times. Response times were measured from the end of the stimulus to the participants' mouse click.

### 2.4. Statistical analysis

All statistical analyses were performed in R (R Core Team, 2022). The *lme4* package (Bates et al., 2015) was used to build mixed statistical models, and the *lmerTest* package (Kuznetsova et al., 2017) was used to obtain *p*-values for the models. Details of the fixed effects are discussed along with the results in the following section. All models included random intercepts for listener (participant), speaker (of the stimuli), and stimulus word.

## 3. Results

### 3.1. Overall categorization

**Figure 1:**
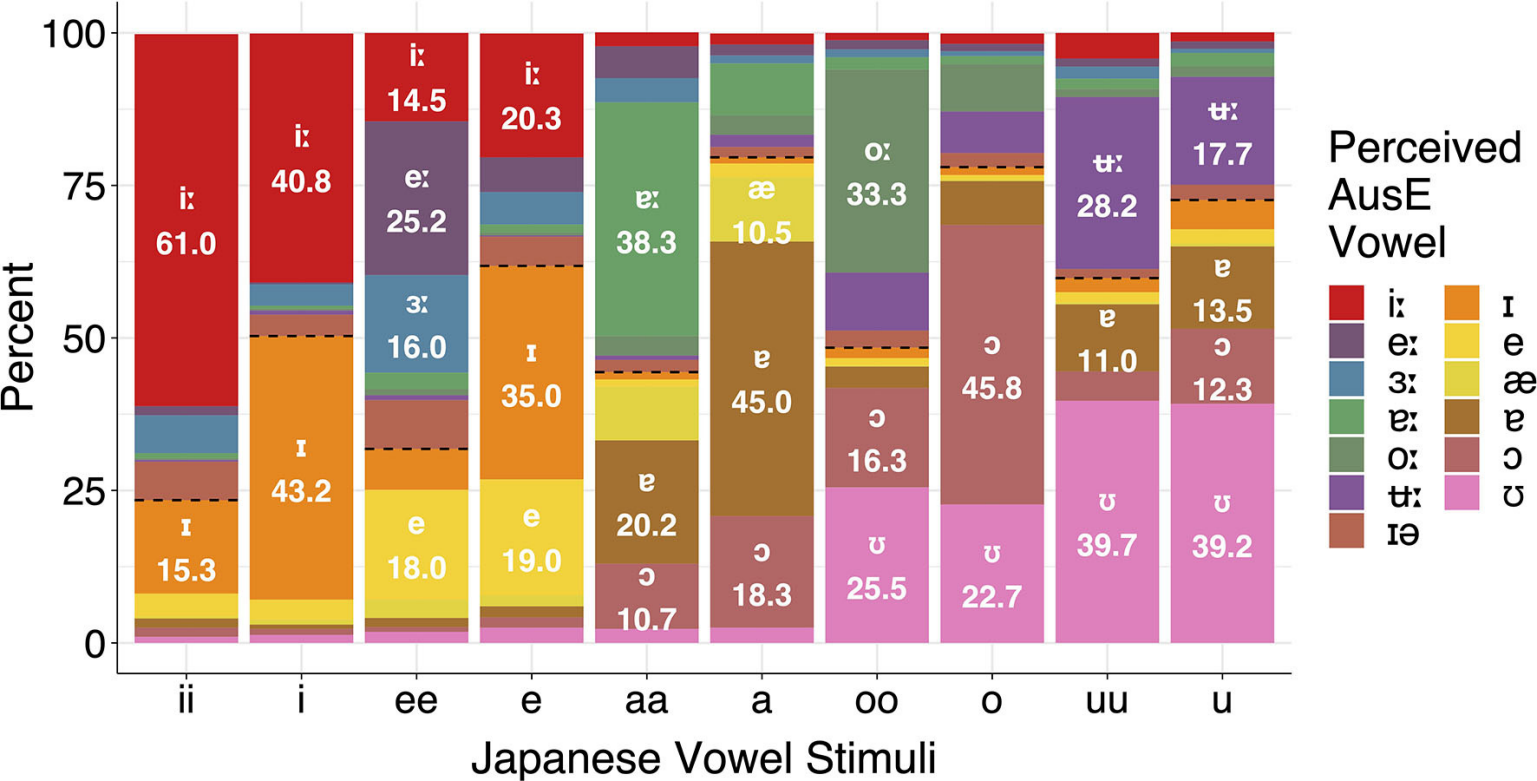
Categorization of Japanese vowels by all participants. The dotted lines show the boundaries between “long” and “short” AusE responses. Only responses >10% are labeled.

Figure 1 presents the overall response patterns. The question going into the experiment was whether AusE listeners' categorization would be affected by Japanese vowel length and, if so, whether and how the effect would be related to Japanese vowel quality. To answer this question, both the Japanese target vowels and AusE response vowels were first collapsed by length (“long” vs. “short”).^5^ A generalized linear mixed model (GLMM) with a logit link function was then fitted using the *glmer()* function, with AusE vowel length (1 = “long,” 0 = “short”) as the outcome variable and Japanese vowel length (“long,” “short”), Japanese vowel quality (/i, e, a, o, u/), and their interaction as the predictor variables. The predictors were coded with sum contrast coding so that each level of a variable is compared to the grand mean rather than a fixed reference level.

Table 3 presents the results of the analysis. Note that the table shows the combined results of two models of the same structure but with different reference levels. This is because regression models do not return the coefficient of the reference level, and although the missing coefficient can be calculated by hand, its significance level is not examined, making a second model necessary (Clopper, 2013). Changing the reference level results in negligible changes in the coefficients for the non-reference levels, and thus for viewing convenience, Table 3 combines (a) the result of a model with Japanese /u/ as the reference level and (b) the result for Japanese /u/ obtained from a model with Japanese /i/ as the reference level.^6^

**Table 3:** GLMM analysis on effects of Japanese length and quality on AusE length categorization.

	**β**	**SE**	***z***	***p*-value**	
(Intercept)	−0.241	0.128	−1.876	0.060	.
long	0.630	0.063	10.017	2^−16^	^***^
/i/	0.865	0.126	6.864	7^−12^	^***^
/e/	0.382	0.125	3.059	0.002	^**^
/a/	−0.364	0.126	−2.884	0.004	^**^
/o/	−0.394	0.126	−3.133	0.002	^**^
/u/	−0.489	0.125	−3.905	9^−05^	^***^
long:/i/	0.011	0.126	0.086	0.932	
long:/e/	0.034	0.125	0.270	0.787	
long:/a/	0.212	0.126	1.676	0.094	.
long:/o/	0.072	0.126	0.569	0.569	
long:/u/	−0.328	0.125	−2.618	0.009	^**^

Baseline = grand mean (^***^ = 0.001, ^**^ = 0.01, * = 0.05, . = 0.1).

The main effect of Japanese length (i.e., “long”) was statistically significant, suggesting that AusE listeners tended to choose “long” AusE response categories when the target Japanese vowel was phonologically long. The main effect of Japanese quality was also all significant, indicating that the likelihood of “long” AusE response categories being chosen differed according to the target Japanese quality. This is expected, as the number of AusE “long” and “short” vowels that correspond to a Japanese quality can vary depending on the quality, as can be seen in Figure 1. In contrast, the interaction between Japanese length and quality was significant only for /u/. This indicates that the effect of Japanese length on AusE listeners' categorization was generally independent of Japanese quality, except for /u/. The negative coefficient of the significant interaction implies that listeners tended to choose a “short” AusE vowel when the target Japanese vowel was long (i.e., /uu/).

### 3.2. By-vowel categorization

**Figure 2:**
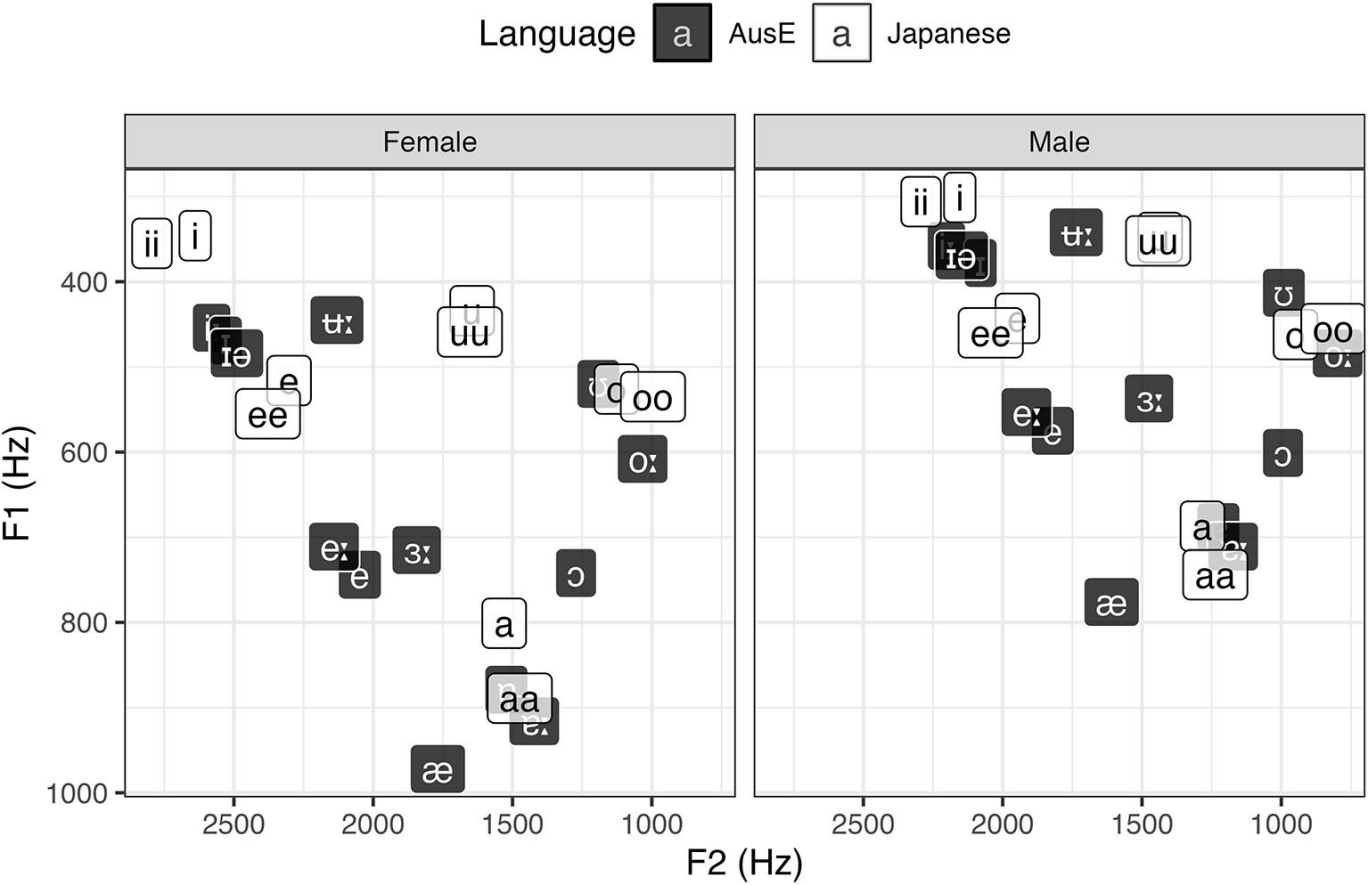
Average F1 and F2 of AusE and Japanese vowels. Adapted from Elvin et al. (2016) and Yazawa and Kondo (2019).

**Figure 3:**
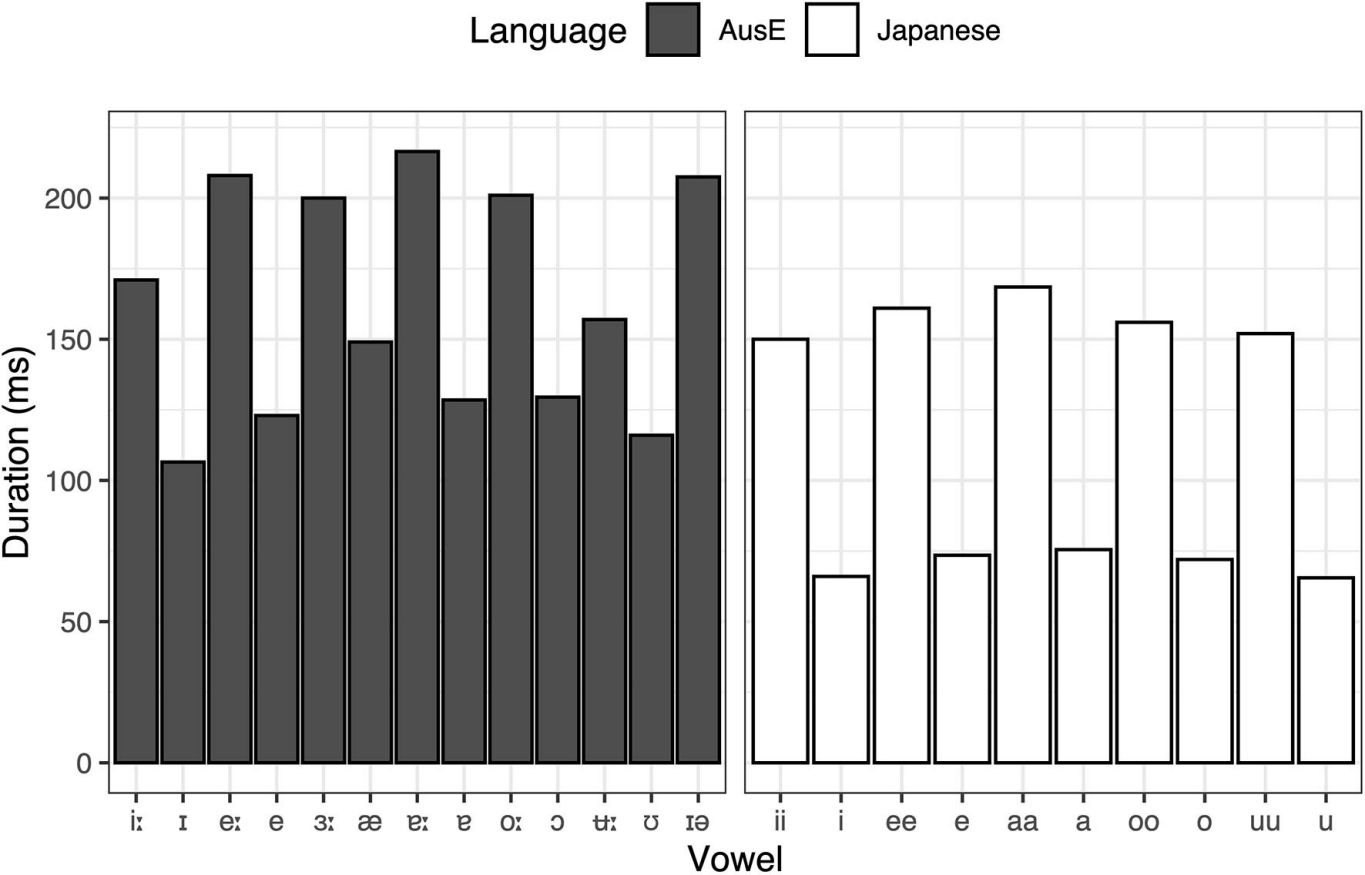
Average duration of AusE and Japanese vowels (male and female means collapsed). Adapted from Elvin et al. (2016) and Yazawa and Kondo (2019).

**Figure 4:**
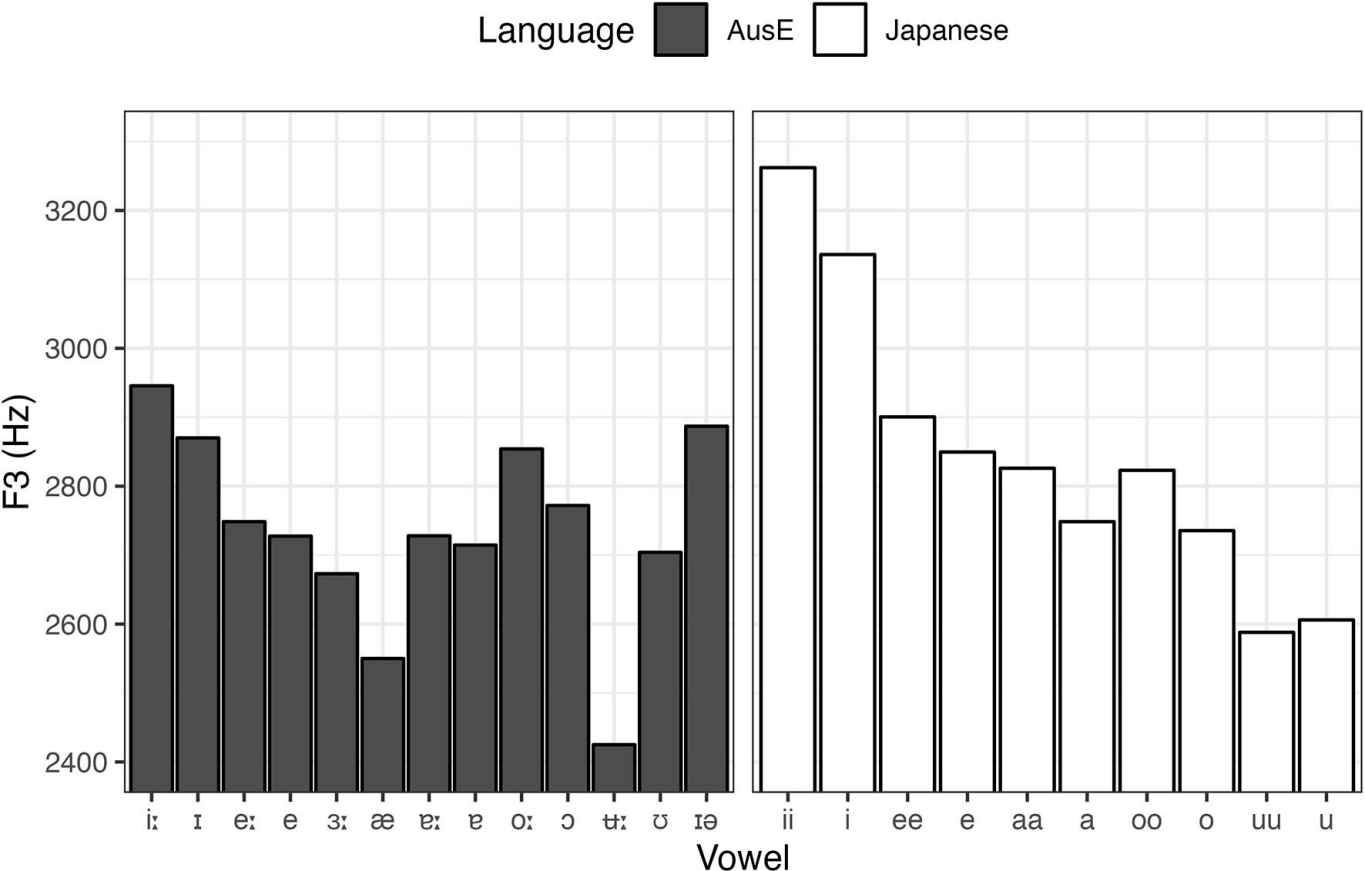
Average F3 of AusE and Japanese vowels (male and female means collapsed). Adapted from Elvin et al. (2016) and Yazawa and Kondo (2019).

In order to explore the factors that drove the overall categorization patterns in more detail, we also performed by-vowel analyses, fitting GLMMs for AusE vowel responses that are the closest to Japanese vowels in terms of height, backness, roundedness, and length. To complement the analyses, acoustic data of the relevant AusE and Japanese vowels (Elvin et al., 2016; Yazawa and Kondo, 2019) are presented in Figures 2, 3, 4.

We start with high vowels, where the front vowels /ii, i/ showed a clear effect of duration on categorization but the back vowels /uu, u/ did not. In the case of Japanese /ii, i/, both vowels were categorized predominantly as their closest counterparts in AusE—high, front, unrounded, long/short—namely /i:/ and /ɪ/. AusE listeners showed a clear preference for AusE /i:/ when categorizing Japanese long /ii/ (61.0%), but were split between /i:, ɪ/ when categorizing Japanese short /i/ (40.8 and 43.2%, respectively). To test whether AusE listeners chose AusE categories that matched to Japanese vowels both in terms of length and quality, we first fitted a GLMM with logit link function to the whole data, with the rate of AusE /i:/ responses (1 = /i:/ chosen, 0 = /i:/ not chosen) as the outcome variable and Japanese vowel category (/ii, ee, aa, oo, uu, i, e, a, o, u/) as the predictor. Japanese /ii/ was set as the baseline, which was expected to have the highest AusE /i:/ response rates across all ten Japanese vowels. The analysis found that AusE listeners categorized Japanese /ii/ significantly more often as AusE /i:/ than Japanese /i/ (β = −0.884, *SE* = 0.297, *t* = −2.974, *p* = 0.003) as well as all other Japanese vowels (*ps* < 0.001). To assess whether a similar length-based difference is found for vowels categorized as AusE /ɪ/, another GLMM of the same structure was fitted for AusE /ɪ/ responses with Japanese /i/ as the baseline. The results showed that AusE listeners categorized Japanese /i/ significantly more often as AusE /ɪ/ than both Japanese /ii/ (β = −1.609, *SE* = 0.411, *t* = −3.910, *p* < 0.001) and all other vowels (*ps* < 0.001), with the exception of /e/ (β = 0.367, *SE* = 0.402, *t* = −0.913, *p* = 0.361). Since spectral differences between Japanese /ii, i/ are negligible, the two vowels should show similar categorization patterns if AusE listeners were relying primarily on quality, much like AmE listeners in Nishi et al. (2008). This is clearly not the case, where instead AusE listeners make use of the longness of Japanese /ii/ to categorize it as AusE /i:/. On the other hand, the shortness of Japanese /i/ does not seem to have an effect.

Similar to Japanese /ii, i/, AusE listeners also categorized Japanese /uu, u/ predominantly as their closest AusE vowels in terms of height and backness—high, non-front—namely AusE /ʉ:, ʊ/, respectively. However, while duration did have a significant effect on how the Japanese vowels were categorized, the effect was relatively small compared to Japanese /ii, i/ in that both Japanese /uu, u/ were categorized most often as AusE /ʊ/, a short vowel. As with the responses to Japanese /ii, i/, we fitted two GLMMs, one for the rate of AusE /ʉ:/ responses and another for /ʊ/ responses, to test whether AusE listeners chose AusE categories that matched to Japanese vowels in terms of length. The model for AusE /ʉ:/ responses indeed showed that Japanese /uu/ was categorized more often as AusE /ʉ:/ than Japanese /u/ (β = −0.679, *SE* = 0.218, *t* = −3.117, *p* = 0.002) and all other vowels (*ps* < 0.001). However, there was no significant difference between Japanese /uu/ and /u/ among vowels categorized as AusE /ʊ/ (β = 0.026, *SE* = 0.200, *t* = 0.131, *p* = 0.896), showing that both long and short Japanese vowels were equally likely to be categorized as AusE /ʊ/. A possible factor driving the observed pattern is roundedness. According to Harrington et al. (1997) and Cox (2006), AusE /ʉ:/ often exhibits onglide with lowering third formant (F3), i.e., increased lip rounding toward the target. This is shown in Figure 4, where AusE /ʉ:/ shows unusually low F3 that is much lower than that of AusE /ʊ/ and Japanese /uu, u/. Since both AusE /ʊ/ and Japanese /uu, u/ lack such articulation, AusE listeners might have prioritized the lack of lowered F3 itself as a cue for AusE /ʊ/ over the long duration as a cue for AusE /ʉ:/.^7^

The remaining Japanese vowels all patterned more closely with Japanese /ii, i/ than with /uu, u/ in that long Japanese vowels clearly led to more categorizations as long AusE vowels. In the case of Japanese /ee, e/, AusE listeners categorized /ee/ as a long AusE vowel 68.2% of the time and /e/ as short 61.8% of the time. GLMMs were fitted for AusE /e:, e/ responses, vowels closest to Japanese /ee, e/ in terms of height, backness, and roundedness. The model for AusE /e:/ responses showed that Japanese /ee/ was significantly more likely to be categorized as the AusE vowel than all other Japanese vowels (*ps* < 0.001). Likewise, the model for AusE /e/ responses also showed that Japanese /e/ was significantly more likely to be categorized as the AusE vowel than all other Japanese vowels (*ps* < 0.001), except for /ee/ (β = −0.070, *SE* = 0.193, *t* = −0.361, *p* = 0.718). One thing to note is that both Japanese /ee, e/ were categorized persistently as AusE /e/, a short vowel, at rates of 18.0 and 19.0%, respectively, and AusE /i:/, a long vowel, at rates of 14.5 and 20.3%, respectively. The persistence of /e, i:/ categorizations for both vowels seems to reflect listeners' uncertainty regarding the spectral quality of Japanese /ee, e/, which lie between the AusE high and mid front vowels (Figure 2). This explains why the GLMM for AusE /ɪ/ responses did not yield a significant difference between Japanese /i/ and /e/ as mentioned above. Therefore, the assumption that Japanese /ee, e/ should be categorized predominantly as AusE /e:, e/, which contrast primarily in duration unlike most other AusE vowels and may thus elicit an elevated duration effect, may not necessarily hold. Despite the increased variability in categorization, however, the effect of length largely parallels the pattern observed with Japanese /ii, i/; AusE listeners use the longness of Japanese /ee/ to categorize it as an AusE long vowel and the shortness of Japanese /e/ to categorize it as an AusE short vowel.

For the low Japanese vowels, the most frequent response category for Japanese long /aa/ was AusE /ɐ:/ (38.3%) and for Japanese short /a/ was AusE /ɐ/ (45.0%), showing again a duration-based preference according to height, backness, and roundedness. However, like AusE /e:, e/ discussed above, AusE /ɐ:, ɐ/ are spectrally overlapping and thus contrast primarily in duration, which admittedly makes the duration effect in the categorization of Japanese /aa, a/ seem weaker than expected (since the effect is comparable to that in the categorization of Japanese /ii, i/ as AusE /i:, ɪ/ which contrast both spectrally and durationally). This is perhaps due to the fact that Japanese /aa/ is durationally ambiguous between AusE /ɐ:/ and /ɐ/, as can be seen in Figure 3. This ambiguity effect is actually reflected by the fact that the second-most frequent categorization of Japanese /aa/ was AusE /ɐ/ (20.2%). Despite the seemingly weak duration effect, the results of GLMMs nonetheless showed that Japanese /aa/ and /a/ were significantly more likely to elicit AusE /ɐ:/ and /ɐ/ responses, respectively, than all other Japanese vowels (*ps* < 0.001).

Lastly, the categorization pattern is similar with Japanese /oo, o/, where the vowels were significantly more likely to elicit AusE /o:/ and /ɔ/ responses, respectively, according to GLMMs (*ps* < 0.001). However, both Japanese /oo, o/ also showed rather persistent categorizations as AusE /ʊ/ regardless of length (25.5 and 22.7%, respectively). This is most likely due to the spectral uncertainty of Japanese back /oo, o/ as either AusE high back /ʊ/ or non-high back /o:/. It is noteworthy, therefore, that Japanese /o/ was most often categorized as AusE /ɔ/ despite the spectral mismatch, suggesting that durational similarity (i.e., shortness) was prioritized over spectral similarity (i.e., height).

### 3.3. Response time

The response time data were analyzed with a linear mixed-effects model (LME). The model was fitted using the *lmer()* function, with response time (in seconds) as the outcome variable and Japanese vowel category (/ii, ee, aa, oo, uu, i, e, a, o, u/) as the predictor variable. The predictor was again sum contrast coded to set the baseline of the model as the grand mean. The results are presented in Table 4, which combines (c) the result of a model with Japanese /u/ as the reference level and (d) the result for Japanese /u/ obtained from a model with Japanese /ii/ as the reference level, for the same reason as stated in Section 3.1.

**Table 4:** LME analysis comparing response times by Japanese vowel category.

	**β**	**SE**	***z***	***p*-value**	
(Intercept)	3.166	0.224	14.110	4.52^−12^	^***^
/ii/	−0.388	0.095	−4.063	4.91^−05^	^***^
/i/	−0.303	0.095	−3.177	0.001	^**^
/ee/	0.257	0.095	2.696	0.007	^**^
/e/	0.400	0.095	4.193	2.79^−05^	^***^
/aa/	0.166	0.095	1.740	0.082	.
/a/	−0.057	0.095	−0.594	0.553	
/oo/	0.292	0.095	3.057	0.002	^**^
/o/	−0.183	0.095	−1.919	0.055	.
/uu/	−0.121	0.095	−1.269	0.205	
/u/	−0.063	0.095	−0.664	0.506	

Baseline = grand mean (^***^ = 0.001, ^**^ = 0.01, ^*^ = 0.05, . = 0.1).

The results show that listeners took the shortest to categorize Japanese /ii/ at 2.778 s and Japanese /i/ at 2.863 s, which are both significantly shorter than the grand mean of 3.166 s. This suggests that the Japanese /i/ quality was relatively easy to categorize, probably because it is unambiguously high and front. In contrast, listeners took significantly longer than the grand mean to categorize Japanese /e/ at 3.566 s and Japanese /ee/ at 3.423 s. This suggests that the Japanese /e/ quality was generally difficult to categorize, most likely due to its spectral ambiguity as discussed earlier. Another Japanese vowel that took significantly longer than the grand mean was /oo/, probably due to its ambiguous quality between AusE /o:/ and /ʊ/. It is then worth noting that the response time for Japanese /o/ was marginally shorter than the grand mean (−0.183 s, *p* = 0.055), as it implies that Japanese /o/ was less ambiguous than Japanese /oo/ despite both vowels being spectrally alike, suggesting that the short duration of /o/ outweighs the spectral ambiguity.

One additional factor that is relevant to the response time data is potential lexical effects. While listeners were instructed that the stimuli were not English words, some of the Japanese tokens (e.g., /biip/) that resemble a real English word (e.g., *beep*) may have implicitly activated AusE lexical knowledge. Since listeners used word choices (e.g., *heed*) to respond, such tokens may have been processed faster than other tokens with no corresponding English word (e.g., /gaak/). To test this possibility, listeners' responses were coded as either “lexical” or “non-lexical,” where “lexical” responses have a corresponding AusE lexical item. For example, if a listener chose AusE /i:/ when the target stimulus's consonantal context was /bVp/, the response was coded as “lexical” because the perceived form /bi:p/ corresponds to a real AusE word *beep*. Other cases of “lexical” responses were: /ɔ/ responses to /bVp/ stimuli (i.e., *bop*), /e, ɐ:, ɔ/ responses to /dVt/ stimuli (i.e., *debt, dart, dot*, respectively), and /i:, o:, ʉ:/ responses for /gVk/ stimuli (i.e., *geek, gawk, gook*, respectively). The remaining responses were coded as “non-lexical,” which accounted for 72.8% of all responses (4,370 of 6,000).

Adding this variable of lexicality with sum contrast coding to the aforementioned LME model significantly improved the model fit according to a likelihood ratio test [χ^2^(1) = 14.585, *p* < 0.001]. The resultant model found a significantly shorter response time for “lexical” responses than “non-lexical” ones (β = −0.136, *SE* = 0.037, *t* = −3.636, *p* < 0.001). Thus, it is speculated that AusE listeners recognized English words in some of the Japanese tokens, which were processed faster than the other tokens without any lexical reference. Yet, further addition of the interaction of lexicality and Japanese vowel category did not improve the model fit [χ^2^(9) = 10.361, *p* = 0.322], meaning that there were no by-category differences in the shortening effect of lexicality on response time.^8^

## 4. Discussion

### 4.1. Summary of the results

The first purpose of this study was to evaluate whether vowel duration is used phonologically in AusE, unlike most other varieties of English. Previous research had shown that AusE listeners are sensitive to acoustic-phonetic changes in vowel duration (Tsukada, 2012), but it was unclear whether they would actively utilize the duration cue for their native vowel identity. The current study therefore examined AusE listeners' categorization of Japanese long and short vowel pairs, each of which differs systematically in duration but minimally in spectral quality (cf. Figures 2, 3, 4). The analysis found a general tendency for Japanese long and short vowels to be categorized as AusE long and short vowels, respectively (Figure 1 and Table 2), indicating that vowel duration does play an important role in AusE phonology. The result contrasts with previously reported AmE listeners' categorization of the same Japanese vowels (Nishi et al., 2008), which was largely unaffected by length (Table 1).

The second purpose was to test whether the above effect of duration on AusE listeners' vowel categorization would be specific to certain Japanese categories or generalized across the board. Given that only a subset of AusE vowels contrast in duration alone (/e:/-/e/ and /ɐ:/-/ɐ/) while others contrast in both duration and spectra (e.g., /i:/-/ɪ/ and /o:/-/ɔ/), AusE listeners may use duration more readily for Japanese vowels that spectrally match the former categories (e.g., /ee/-/e/ and /aa/-/a/) than those matching the latter (e.g., /ii/-/i/ and /oo/-/o/). However, the AusE listeners in the current study seem to have utilized duration for both cases regardless of spectral ambiguity (Table 4), suggesting that the effects of length and quality were generally independent of each other, with a notable exception of /uu/-/u/ (Table 3). These results have important theoretical and pedagogical implications, as discussed below.

### 4.2. Theoretical implications

As outlined in Section 1, the segment- and feature-based frameworks of speech perception predicted different results for the current experiment. On the one hand, the segment-based view predicted that the effect of duration should be stronger for certain Japanese qualities (i.e., /e, a/) than the others (i.e., /i, o, u/), as the reliance on duration cues should be specific to each native segmental category that nonnative sounds are categorized as. On the other hand, the feature-based view predicted a uniform effect of duration across all Japanese categories, assuming that a length feature plays a role in the whole phonological system. The GLMM analysis in Table 3 suggests that the observed perceptual patterns align better, but not perfectly, with the feature-based view. Vowel length had an independent effect from vowel quality, where long Japanese vowels tended to be categorized as long AusE vowels despite mismatches in quality. In this respect, the categorization tendency was largely the same between AusE vowels that contrast in both duration and spectra (e.g., /i:, ɪ/) and those that contrast exclusively in duration (e.g., /ɐ:, ɐ/). The only exception was Japanese /uu, u/, which were consistently categorized as short AusE /ʊ/.

Importantly, this kind of generalization of native length to nonnative perception has been observed in other previous studies as well. Returning to Tsukada (2012)'s study, Arabic differs from Japanese in lacking the /e/ and /o/ qualities and, according to the segment-based view, native Arabic listeners should be less accurate in discriminating the length of Japanese /ee/-/e/ and /oo/-/o/ (absent segments) than /ii/-/i/, /aa/-/a/, and /uu/-/u/ (present segments). The result contrarily showed no difference in discrimination ability between present and absent qualities, which is in line with the feature-based view. The segment-based view would also have difficulty in explaining the link between vowel and consonant length found in McAllister et al. (2002) and Pajak and Levy (2014) because it would be implausible for consonant categories to assimilate to vowel categories and vice versa. Moreover, Tsukada et al. (2018) found that both L1 AusE and L1 Korean learners of L2 Japanese were generally accurate in identifying the consonantal length of Japanese as well as Italian (>80%), despite the fact that AusE does not have a singleton-geminate contrast while Korean does. This would support the view that AusE does have a vowel length feature that can transfer or extend to nonnative consonant length perception.

One caveat with the feature-based approach is that the property of the “same” feature can vary from language to language, despite the traditional belief that features are language-universal. As for the vowel length feature, what is “long” in one language is not necessarily also “long” in another language and vice versa, as the actual duration of “long” vowels can differ substantially across languages. This can be seen in Figure 3, where Japanese vowels are shorter in duration than AusE vowels in general. It follows that some tokens of “long” Japanese vowels are not sufficiently long in duration to be categorized as “long” in AusE, which likely affected the categorization patterns shown in Figure 1.^9^ This explanation would align with a recent proposal that features are substance-free and emergent (Boersma et al., 2022); there is no innate phonological substance of absolute “longness” in the mind, and listeners rather learn to interpret what is meaningfully long or short in the given language based on the available linguistic input.

The exceptional categorization pattern of Japanese /uu, u/ by AusE listeners, however, poses a challenge to the feature-based view. As mentioned earlier, the result can only be explained by referring to the F3, an acoustic cue for lip rounding. One may thus hypothesize that a roundedness feature was contributing somehow, although it would still be unclear why only this feature suppressed the effect of length while other features such as height and backness did not. A possible reason lies in the multiplicity of acoustic cues or the lack thereof in the given features. While height, backness, and length features are considered to have only one corresponding acoustic cue (i.e., F1, F2, and duration, respectively), the roundedness feature is associated with multiple acoustic cues (i.e., F2 and F3). Llompart and Reinisch (2018) found that effects of selective adaptation on German vowel contrasts generalized for contrasts differing in height (F1) and those differing in backness (F2) but not for those differing in tenseness (F1, F2, and duration),^10^ suggesting that acoustically complex features such as tenseness and roundedness may behave differently from acoustically simple features such as height, backness, and length in vowel perception.^11^ Assuming that perceptual input is gradually abstracted and integrated into higher-level representations (Greenberg and Christiansen, 2019), it may be the case that acoustically complex features outweigh lower-level, acoustically simple features, which may explain why Japanese /uu/ without strong lip rounding would not be categorized as AusE /ʉ:/ despite their similar durations.

Finally, it should be noted that the dichotomy of segment- vs. feature-based views is not an absolute one. Incorporation of these two approaches is possible, as indicated in the above explanation of gradual abstraction and integration of cues to segments via features. An example of such integration comes from the Second Language Linguistic Perception (L2LP) model (Escudero and Yazawa, in press; Escudero, 2005; van Leussen and Escudero, 2015), which defines speech perception as the mapping of acoustic cues onto a linguistic representation. While the majority of studies conducted within L2LP have assumed segmental categories as the fundamental unit of perception, some studies have referred to other units including features. For example, Escudero and Boersma (2004) demonstrated that L1 Spanish listeners' over-reliance on duration in perceiving the /i:/-/ɪ/ in L2 Southern British English (SBE) can be accurately modeled by assuming that the SBE vowels are represented as /i, long/ and /i, short/ in the learners' phonological grammar, i.e., addition of a new length feature to an existing segmental category. Yazawa (2020) also proposed that Japanese listeners' perception of AmE /æ/ as a deviant, non-prototypical exemplar of Japanese /a/ or possibly /e/ (Strange et al., 1998; Shinohara et al., 2019, 2022) can be explained as a result of mismatch in height and frontness features, i.e., AmE /low, front/ (/æ/) is too front to be Japanese /low, central/ (/a/) and too low to be Japanese /mid, front/ (/e/). An important side note on these studies is that the learners' target variety (SBE or AmE) was explicitly specified, as is proposed within the L2LP model, which is essential for making accurate predictions and explanations regarding cross-linguistic perception patterns (cf. Section 4.4).

### 4.3. Pedagogical implications

Some pedagogical implications for English listeners' learning of nonnative length arise from the above discussion on features. According to the “feature” hypothesis, nonnative length contrasts would be less of a challenge for AusE listeners who has access to a vowel length feature than for AmE speakers who do not.^12^ This prediction has been attested in previous studies showing that monolingual AusE listeners could already discriminate Japanese vowel length well (75% accurate; Tsukada, 2012) while AmE listeners prior to perceptual training identified Japanese vowel length poorly (39% accurate; Hirata, 2004). However, the presence of a vowel length feature in AusE does not guarantee immediate and successful learning of nonnative length contrasts because, as discussed earlier, the acoustic property of a feature is likely language-specific. For example, “long” Japanese vowels that are about 150 ms long can be ambiguous between “long” (200 ms) and “short” (100 ms) for AusE listeners, resulting in occasional misperception of “long” as “short.” This kind of mismatch in featural properties would explain, at least in part, why nonnatives perform consistently worse than natives in vowel and consonant length perception even when they share the “same” feature of length (Tsukada, 2012; Tsukada et al., 2018). The learning task for AusE learners of Japanese, therefore, is to shift the boundary between “long” and “short” vowels to match that of Japanese (which L2LP calls a “perceptual task”). On the other hand, AmE learners of Japanese have an additional task to establish a new length feature in their phonological grammar (a “representational task” in L2LP's term), similar to Spanish learners of SBE as mentioned above. Thus, the presence or absence of a vowel length feature in the two varieties of English leads to different kinds of learning tasks.

The remaining question, then, is how the learning process can be facilitated in such a way that is appropriate for each language variety (Elvin and Escudero, 2019). On the one hand, AusE learners of Japanese may be able to shift their perceptual boundary via simple distributional learning (i.e., abundant exposure to Japanese long and short vowels), perhaps aided by artificially enhanced durational distributions (Escudero et al., 2011), to achieve immediate and long-lasting learning effects (Escudero and Williams, 2014). On the other hand, AmE learners of Japanese may need to be directed to the presence of vowel length more explicitly, as acquiring a new feature seems more problematic than shifting an existing boundary (Chládková et al., 2022). Hirata (2004)'s success in training AmE listeners on Japanese length contrasts may be attributed to the unique training procedure, where AmE participants were instructed to count the number of morae in each training token, e.g., /ii/ “good” (= 2 morae) and /i/ “stomach” (= 1 mora). The participants were thus made aware of Japanese length throughout the training period of 3.5 weeks, potentially resulting in efficient and robust learning. Moreover, the training also involved consonant length, e.g., /kata/ “shoulder” (= 2 morae) and /katta/ “won” (= 3 morae). Given that vowel and consonant length seems interrelated, perhaps the training on vowels and consonants interacted with each other, further enhancing the learning efficacy.^13^ This kind of explicit training could be useful for teaching nonnative length to native listeners of AmE and other varieties of English. What still needs testing is whether the learning task of boundary shift for AusE listeners is really as easy as expected and, if so, how long and to what extent the learning effect can be maintained. To this end, implicit training paradigms such as cross-situational word learning (CSWL) can be useful (Escudero et al., 2022, 2023; Escudero and Yazawa, in press).

### 4.4. Future directions

The current study has demonstrated that AusE listeners systematically utilize duration cues for vowel identity, despite the common belief that length is not phonemic in English. The result contrasts with previous studies on AmE listeners (Hillenbrand et al., 2000; McAllister et al., 2002; Hirata, 2004; Dietrich et al., 2007; Kondaurova and Francis, 2008; Mugitani et al., 2009), especially that of Nishi et al. (2008) who examined the categorization of Japanese long and short vowels. However, the current result cannot be directly compared with that of Nishi et al. (2008) due to methodological differences. For example, while Nishi et al. (2008) used /hVba/ disyllables as stimuli, the current study used varying consonantal contexts (/bVp, gVk, dVt/), but without /hVb/. Based on the large body of previous research reviewed earlier, we can assume with some confidence that AmE listeners would show a similar perceptual pattern to Table 1 with our stimuli and procedure, but an additional parallel data collection in the US would be ideal to allow for a more direct comparison.

The results of the current study also highlight the necessity to further investigate non-AmE varieties of English. Although Karpinska et al. (2015) found similar perceptual trends in high front vowel identification across several varieties of English (except for AusE), these varieties may actually show some variability in perceptual patterns. For example, Escudero and Boersma (2004) showed that SBE listeners rely systematically more on duration than Scottish English listeners when perceiving synthetic high front tense and lax vowels (commonly referred to as “long” and “short” vowels in British English). Moreover, it is unclear whether similar perceptual tendencies would be observed for non-high-front vowels as well, as AmE listeners' reliance on duration seems to somewhat differ between high and non-high vowel contrasts (Hillenbrand et al., 2000), which is reflected in their perception of nonnative Japanese long and short vowels (Nishi et al., 2008). Thus, cross-examining listeners of different varieties of English in their categorization of Japanese length in future studies—preferably with AmE and AusE as references—would shed further light on whether and to what extent the segment- and feature-based approaches are capable of explaining cross-variety similarities and differences.

## Data availability statement

The raw data supporting the conclusions of this article will be made available by the authors, without undue reservation.

## Ethics statement

The present study involving human participants was part of a larger study reviewed and approved by Western Sydney University Human Research Ethics Committee (approval number: H11022). The studies were conducted in accordance with the local legislation and institutional requirements. The participants provided their written informed consent to participate in this study.

## Author contributions

KY and PE contributed to the conception and design of the study. KY collected the production data and wrote the first draft of the manuscript. JW supervised the collection of the perception data and was responsible for the data analysis. JW and PE wrote parts of the manuscript. All authors contributed to the article and approved the submitted version.

## Footnotes

^1^Japanese has five distinct qualities /i, e, a, o, u/, which form five short (1-mora) and long (2-mora) pairs (Keating and Huffman, 1984). The long vowels can be considered phonologically as a sequence of two short vowels and therefore are transcribed with double letters (/ii, ee, aa, oo, uu/) in this paper.

^2^In this study, AusE vowels with and without the “:” symbol are treated as being long and short, respectively.

^3^While the traditional binary feature of length (i.e., [±long]) would not hold here, the obtained results are not incompatible with the feature-based view per se because features can be univalent (Gussenhoven and Jacobs, 2017); segments without a “long” feature are simply unmarked rather than having a “short” feature.

^4^Although accented and unaccented vowels can have different durations in Japanese (Kozasa, 2004), this study uses accented tokens so that the stimuli conform to the default accent placement pattern for loanwords and nonwords (Kawahara, 2015).

^5^AusE /ɪə/ was coded as “long” for convenience.

^6^The main effect and interactions of “short” Japanese length are omitted from the table because the estimate magnitudes of a binary factor are identical with just the signs reversed when sum contrast coded.

^7^While AusE listeners have been shown to be sensitive to such dynamic spectral cues (Williams et al., 2018), note that the Japanese stimuli in the current experiment exhibited very little VISC (Yazawa and Kondo, 2019).

^8^Other studies have also found no item effect when nonwords that sound like real words are used in tasks that combine categorization and lexical learning (Escudero et al., 2022, p. 4).

^9^Hirata and Lambacher (2004) found that Japanese listeners can misidentify a short vowel produced at a slow speaking rate as being long because its duration can overlap with that of a long vowel produced at a fast rate. Thus, speech rate is also relevant to the property of “long” and “short” vowels within a language. The current experiment used stimuli at a normal speaking rate to minimize potential rate effects.

^10^The result needs to be interpreted with caution because selective adaptation effects did generalize for lax vowels; it was only for tense vowels that the effects did not generalize. See also Boersma et al. (2022, p. 664) for a possibility that “tense” and “lax” features do not have any phonetic correlate to begin with.

^11^While Llompart and Reinisch (2018) advocate that acoustic cues rather than phonological features drive vowel perception, they also note that the notion of features would be compatible if they are acoustically or phonetically defined. We agree that phonological features should have a phonetic basis (Boersma and Chládková, 2011; Mesgarani et al., 2014; Chládková et al., 2015a), but would also put forward that acoustic cues cannot be substituted for such phonetically grounded phonological features. This is because a purely cue-based approach would not suffice to explain the current result and other perceptual phenomena that can be readily explained by assuming features. See Boersma and Chládková (2011) for further discussion.

^12^While the original SLM (Flege, 1995) was in favor of the “feature” hypothesis, its recent revision (SLM-r; Flege and Bohn, 2021) has replaced it with the “full access” hypothesis, which claims that L2 learners can gain full access to non-L1 features.

^13^Unlike Hirata (2004), Tajima et al. (2008) found limited effects of training on Canadian English speakers' perception of Japanese vowel and consonant length. This can be due to the differences in training procedure (word identification vs. mora counting) and training period (5 days vs. 3.5 weeks) between the two studies.
